# Comparison of the effectiveness of vitrectomy with silicone oil or perfluoropropane tamponade for myopic foveoschisis with foveal detachment

**DOI:** 10.3389/fmed.2025.1602386

**Published:** 2025-09-23

**Authors:** Handong Dan, Dongdong Wang, Zixu Huang, Yizong Liu, Huiming Hou, Yujia Jin, Zongming Song

**Affiliations:** Henan Eye Hospital, Henan Eye Institute, Henan Provincial People’s Hospital, Henan Provincial People’s Clinical Medical School of Zhengzhou University, People’s Hospital of Henan University, Zhengzhou, Henan, China

**Keywords:** myopic foveoschisis, foveal detachment, pars plana vitrectomy, fovea-sparing internal limiting membrane peeling, silicone oil tamponade, perfluoropropane tamponade

## Abstract

**Introduction:**

Vitrectomy with silicone oil or perfluoropropane (C3F8) tamponade is a standard of treatment for myopic foveoschisis with foveal detachment (MFFD). In this study, we compared the pre- and postoperative best-corrected visual acuity (BCVA) and maximum foveal thickness (MaxFT) of patients with MFFD who underwent vitrectomy with silicone oil or C3F8 tamponade.

**Methods:**

All patients underwent comprehensive ophthalmological examinations, including measurement of BCVA, axial measurement, optometry, intraocular pressure, slit-lamp examination, fundus examination, and optical coherence tomography. Patients with MFFD were divided into two groups. All patients underwent with 23-gauge pars plana vitrectomy with fovea-sparing internal limiting membrane peeling, and fluid-air exchange. One group underwent tamponade with silicone oil, whereas the other group underwent tamponade with C3F8. The silicone oil was completely removed upon complete resolution of MFFD. Phacoemulsification with intraocular lens implantation was performed when a lens opacity was noted during vitrectomy or along with silicone oil removal. BCVA and MaxFT were assessed at 1, 3, 6, and 12-months post-operatively. Patients with silicone oil were additionally assessed 3-months after silicone oil removal. All data were calculated using GraphPad Prism.

**Results:**

Forty-one patients with 41 eyes were enrolled in the study. Eighteen eyes were included in the silicone oil group, whereas 23 eyes were included in the C3F8 group. Both groups demonstrated significant improvement in BCVA and MaxFT at 1, 3, 6, and 12-months post-operatively. There was no significant difference in BCVA and MaxFT between both groups post-operatively. Transient ocular hypertension was noted in six and three cases in the silicone oil and C3F8 groups, respectively. One case in the C3F8 group developed a macular hole. There were no other complications in the two groups.

**Conclusion:**

Vitrectomy with fovea-sparing internal limiting membrane peeling, and silicone oil or C3F8 tamponade are effective and practical treatment options for MFFD.

## 1 Introduction

Pathologic myopia is characterized by an error of refraction or axial length greater than 6.00D and 26 mm, respectively, when accompanied by pathologic fundus changes (1, 2), such as atrophy, myopic traction macular disease, and choroidal neovascularization (3, 4). It is one of the main causes of visual impairment and blindness in patients worldwide (5, 6). Pathologic myopia is associated with the following sequelae: myopic foveoschisis, foveal detachment, full thickness macular hole (MH), and macular hole retinal detachment (7). Myopic foveoschisis occurs in 8%–34% of patients with pathologic myopia (8). This condition has gradually increased as more patients with pathologic myopia undergo optical coherence tomography. Myopic traction with foveal detachment (MFFD) is characterized by retinal interlayer dehiscence and foveal detachment. It is a serious complication of pathologic myopia and a poor prognostic indicator of visual function.

Presently, the pathogenesis of MFFD is unclear. It is generally believed that interlayer division of the macular retinal neuroepithelium is caused by axial elongation, resulting in an abnormal vitreoretinal interface (9–11). While early MFFD can be asymptomatic, it can also be associated with destruction of the foveal structure, leading to progressive central vision loss. As such, timely surgical intervention is important. Many approaches are recommended for MFFD. These include procedures, such as vitrectomy and post-scleral reinforcement. Each approach has its advantages and disadvantages (12). Vitrectomy reduces the tractional forces between the vitreous body and retina; however, myopic foveoschisis may still develop in vitrectomized eyes because of increased axial length. It may increase the risk of cataract and macular hole formation. While post-scleral reinforcement surgery may address some of the issues associated with axial length, the procedure is technically difficult. The vortex veins and optic nerve may be injured during the operation. The optimal surgical approach should consider the patient’s condition, and the surgeon’s technical skill. The selected approach should reapproximate the normal anatomy as much as possible and produce the least postoperative complications. Pars plana vitrectomy is currently the primary choice for treating MFFD (13). Vitrectomy works by removing tractional forces from the vitreouretinal interface, which allows the macula to revert to its normal structure.

However, it remains unclear whether vitrectomy with silicone oil or perfluoropropane (C3F8) tamponade is better for patients with MFFD (14). Either approach has its advantages and disadvantages. In this study, patients underwent pars plana vitrectomy with fovea-sparing internal limiting membrane (ILM) peeling and silicone oil or C3F8 tamponade. We compared the best corrected visual acuity (BCVA), maximum foveal thickness (MaxFT), and post-operative complications between two approaches for MFFD to determine which is better and more effective.

## 2 Materials and methods

### 2.1 Subjects

This was a retrospective single-center study. All subjects provided their written informed consent. The study design complied with the tenets of the Declaration of Helsinki and was approved by the ethics committee of Henan Eye Hospital. Consecutive subjects were recruited at Department of Ophthalmology of Henan Eye Hospital from October 2020 to August 2023.

### 2.2 Examinations

All patients underwent comprehensive baseline ophthalmological examinations, which included BCVA, refraction, axial length measurement (IOL-master700, Carl Zeiss Meditec AG, Jena, Thuringia, Germany), slit lamp examination, intraocular pressure, funduscopy examination, and optical coherence tomography imaging. BCVA was measured using the international decimal visual acuity chart, which was then converted to linear data as the logarithmic minimum angle of resolution (log MAR) according to the study. A BCVA score of counting fingers is equivalent to a log MAR value of 2.0, whereas hand movement corresponds with a log MAR value of 3.0 (15). Fundus photography was performed with a VISUCAM 200 digital fundus camera (Carl Zeiss Meditec AG, Jena, Thuringia, Germany) or Optos Daytona ultrawide field system (Optos PLC, Dunfermline, United Kingdom). Optical coherence tomography images were acquired using the SPECTRALIS Engineering system (Heidelberg Engineering Ltd., Hertfordshire, United Kingdom), which captured vertical and horizontal scans of the macular retina. MaxFT was measured as the maximum vertical distance from the retinal pigment epithelium to the internal limiting membrane (ILM) of the foveal retina using the built-in measuring tool of the optical coherence tomography machine.

### 2.3 Inclusion and exclusion criteria

Inclusion criteria: (1) age ≥ 18-years old; (2) high myopia with spherical equivalent ≤ −6.00D or axial length ≥ 26 mm; (3) Optical coherence tomography images showing MFFD; and (4) related symptoms, such as visual loss and metamorphopsia.

Exclusion criteria: (1) other ocular conditions that may affect BCVA and fundus structure, such as macular hole, retinal detachment, macular choroidal neovascularization, diabetic retinopathy, glaucoma, previous vitreous surgery, and eye trauma; (2) incomplete ophthalmologic examination due to opaque refracting media and failure to cooperate; (3) inability to maintain prone position for the required post-operative period; and (4) inability to complete the required follow-up visits.

### 2.4 Surgical procedures

Only one eye from each patient was included in this study. If both eyes met the inclusion criteria, one eye was randomly selected for inclusion in the study. The patients were divided into two groups, and the same surgeon performed all the procedures. All patients underwent with 23-gauge pars plana vitrectomy with the following steps: (1) triamcinolone (0.02 ml, 40 mg/mL) (Kunming Jida Pharmaceutical Corporation, China) was injected into the vitreous cavity to stain the vitreous body and posterior cortex; (2) vitrectomy was performed completely with 23-gauge ports (Stellaris PC, Bausch & Lomb Incorporation, USA); (3) Fovea-sparing internal limiting membrane peeling was done with intraocular microforceps. ILM peeling was limited to the vascular arches and spared the fovea. The entire fundus was also explored intraoperatively. Any retinal holes were sealed with laser photocoagulation, and epiretinal membranes were peeled as needed; and (4) silicone oil (Oxane 5700, Bausch & Lomb Incorporation, USA) or 12% C3F8 (ISPAN Perfluoropropane, Alcon Laboratories Incorporation, USA) was injected depending on the patient’s assigned group. Vitrectomy increases the risk of cataract formation. As such, phacoemulsification with intraocular lens (MA60AC, Alcon Laboratories Incorporation, USA) implantation was performed on patients 50-years old or above and/or when a lens opacity was noted during vitrectomy or along with silicone oil removal. Silicone oil was removed when MFFD was completely recovered according to the previous study (16).

### 2.5 Postoperative treatment

Patients were prescribed post-operative tobramycin and dexamethasone eye drops four times a day, tobramycin and dexamethasone eye ointment once a night, and compounded 0.5% phenylephrine hydrochloride and 0.5% tropicamide eye drops four times a day for 1 month. Intraocular pressure-lowering medications, such as cartelol and brinzolamide eye drops (twice a day), were prescribed when intraocular pressure was higher than 21 mmHg and continued until intraocular pressure stabilized at 15 mmHg. Patients were required to maintain a prone position for at least 12 h daily for 3 weeks.

### 2.6 Postoperative follow-up

Assessment of BCVA, intraocular pressure, MaxFT, and the fundus was done at 1, 3, 6, and 12 months (±2 weeks) post-operatively. Patients who underwent silicone oil removal were followed up additionally after 3 months. Complete recovery was defined as resolution of the retinal interlayer dehiscence and foveal detachment, as assessed on optical coherence tomography. Partial recovery was defined as incomplete resolution of the retinal interlayer dehiscence and macular detachment, characterized by a decrease in the total height of the observed intraretinal space. These patients were scheduled for additional follow-ups. If pathologic progression was noted at any of the follow-ups, a second vitrectomy was performed. Silicone oil removal was performed once the macular architecture was deemed satisfactory. Invalid recovery was defined as a maintenance or worsening of the retinal interlayer dehiscence and macular detachment. These patients underwent a second vitrectomy.

### 2.7 Statistical analysis

All measurement data were tested for normality. Normally distributed measurement data were expressed as mean ± standard deviation. Abnormally distributed measurement data were expressed as quartiles. For normally distributed measurement data, the *T*-test and ANOVA test were performed between two groups and intra-group, respectively. For non-normally distributed measurement data, the Mann-Whitney test and Fridman test were performed between two groups and intra-group, respectively. All enumeration data were represented by examples and component ratios. The chi-squared test was performed for count data. A *P* < 0.05 was considered statistically significant. All data were analyzed using the GraphPad Prism 9 statistical software (GraphPad Software, San Diego, CA, USA).

## 3 Results

### 3.1 Characteristics

**FIGURE 1:**
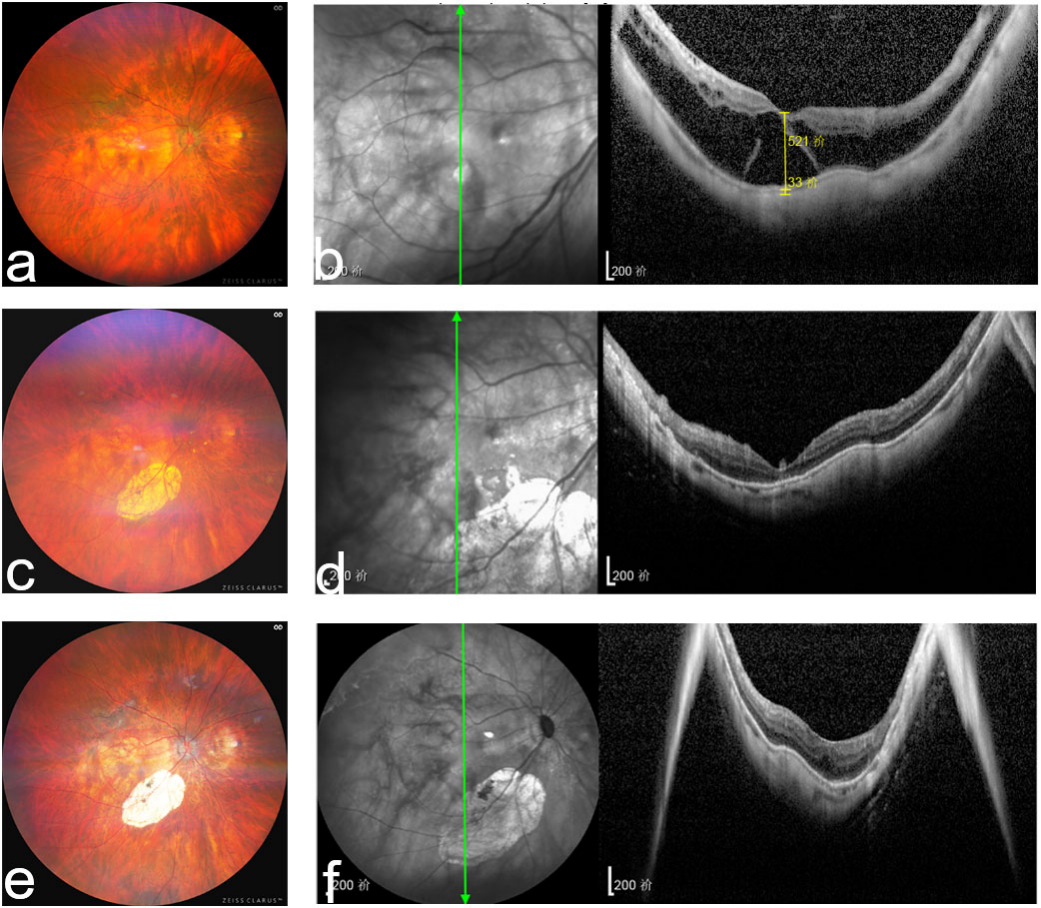
Right fundus and optical coherence tomography images of a representative patient from the silicone oil group. **(a)** Preoperative fundus images showing retinal and choroidal atrophy in a highly myopic patient; **(b)** preoperative optical coherence tomography images showing outer myopic foveoschisis, foveal detachment, and outer macular lamellar hole; **(c)** postoperative fundus images 12 months after vitrectomy with silicone oil tamponade showing retinal and choroidal atrophy. Laser marks can be noted along the inferior vascular arch; **(d)** postoperative optical coherence tomography images showing complete resolution of retinal foveoschisis and foveal detachment; **(e)** postoperative fundus images 3 months after silicone oil removal and phacoemulsification with intraocular lens implantation showing retinal and choroidal atrophy. Laser marks can be noted along the inferior vascular arch; and **(f)** postoperative optical coherence tomography images showing complete resolution of retinal foveoschisis and foveal detachment without recurrence.

**FIGURE 2:**
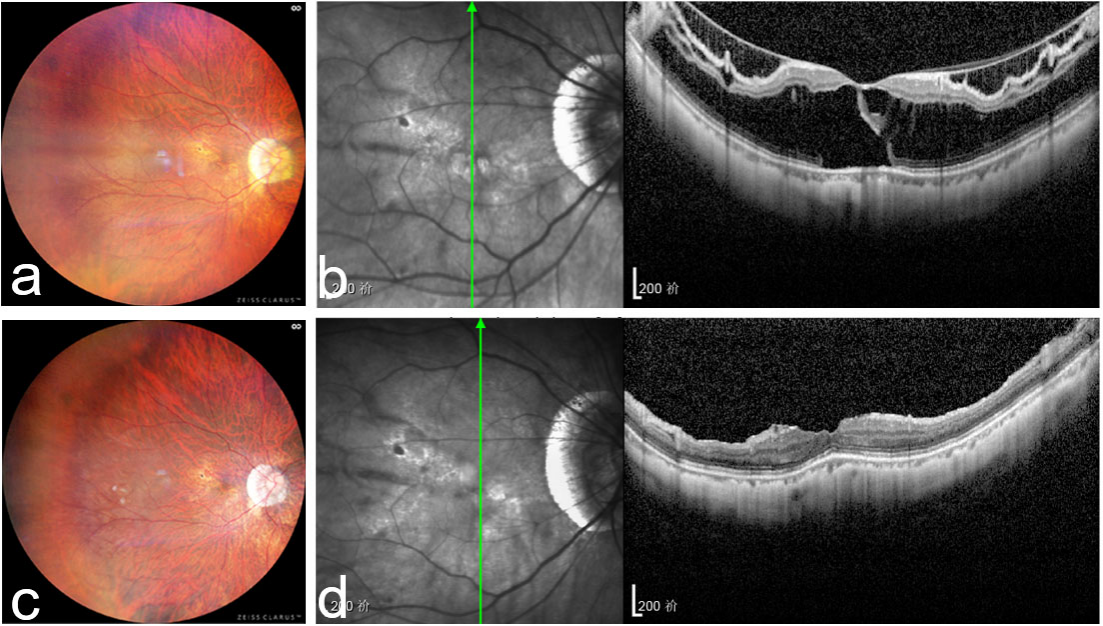
Right fundus and optical coherence tomography images of a representative patient from the perfluorocarbon (C3F8) group. **(a)** Preoperative fundus images showing retinal and choroidal atrophy and an atrophic arc of optic disk; **(b)** preoperative optical coherence tomography images showing outer and inner myopic foveoschisis, foveal detachment, and an outer macular lamellar hole; **(c)** postoperative fundus images 15 months after vitrectomy with C3F8 tamponade showing retinal and choroidal atrophy and an atrophic arc of optic disk; and **(d)** postoperative optical coherence tomography images showing complete resolution of retinal foveoschisis and foveal detachment.

Forty-one patients with 41 eyes were enrolled in the study. The median age was 57.1 ± 6.3 years. Twenty-two (53.7%) participants were men, and 19 (46.3%) were women. Eighteen eyes were included in the silicone oil group, whereas 23 eyes were included in the C3F8 group. There was no statistically significant difference in age, diopter, axial length, intraocular pressure, BCVA, and MaxFT between the two groups. Clinical data of patients with MFFD are summarized in Table 1. The fundus and optical coherence tomography images of a representative patient from the silicone oil group are displayed in Figure 1. The same results for a representative patient from the C3F8 group are displayed in Figure 2.

**TABLE 1:** Clinical data of patients with myopic foveoschisis with foveal detachment.

Characteristic	Silicone oil group	C3F8 group	Test value	*P*-value
Total eye number	18	23	NA	NA
Eye number of phacoemulsification with intraocular lens implantation	17	21	NA	NA
Age	56.6 ± 6.4	57.8 ± 6.2	*T* = 0.614	0.543
Diopter (D)	−10.9 ± 3.5	−10.5 ± 3.4	*T* = 0.371	0.713
Axial length (mm)	28.3 ± 1.3	28.7 ± 1.3	*T* = 0.986	0.330
Intraocular pressure (mmHg)	16.1 ± 3.5	16.5 ± 3.0	*T* = 0.465	0.645
BCVA (Log MAR)	1.19 ± 0.441	1.22 ± 0.445	*T* = 0.165	0.870
MaxFT (um)	580 ± 72.1	565 ± 77.2	*U* = 181.5	0.5112

BCVA, best corrected visual acuity; MaxFT, maximum foveal thickness.

### 3.2 BCVA change

**FIGURE 3:**
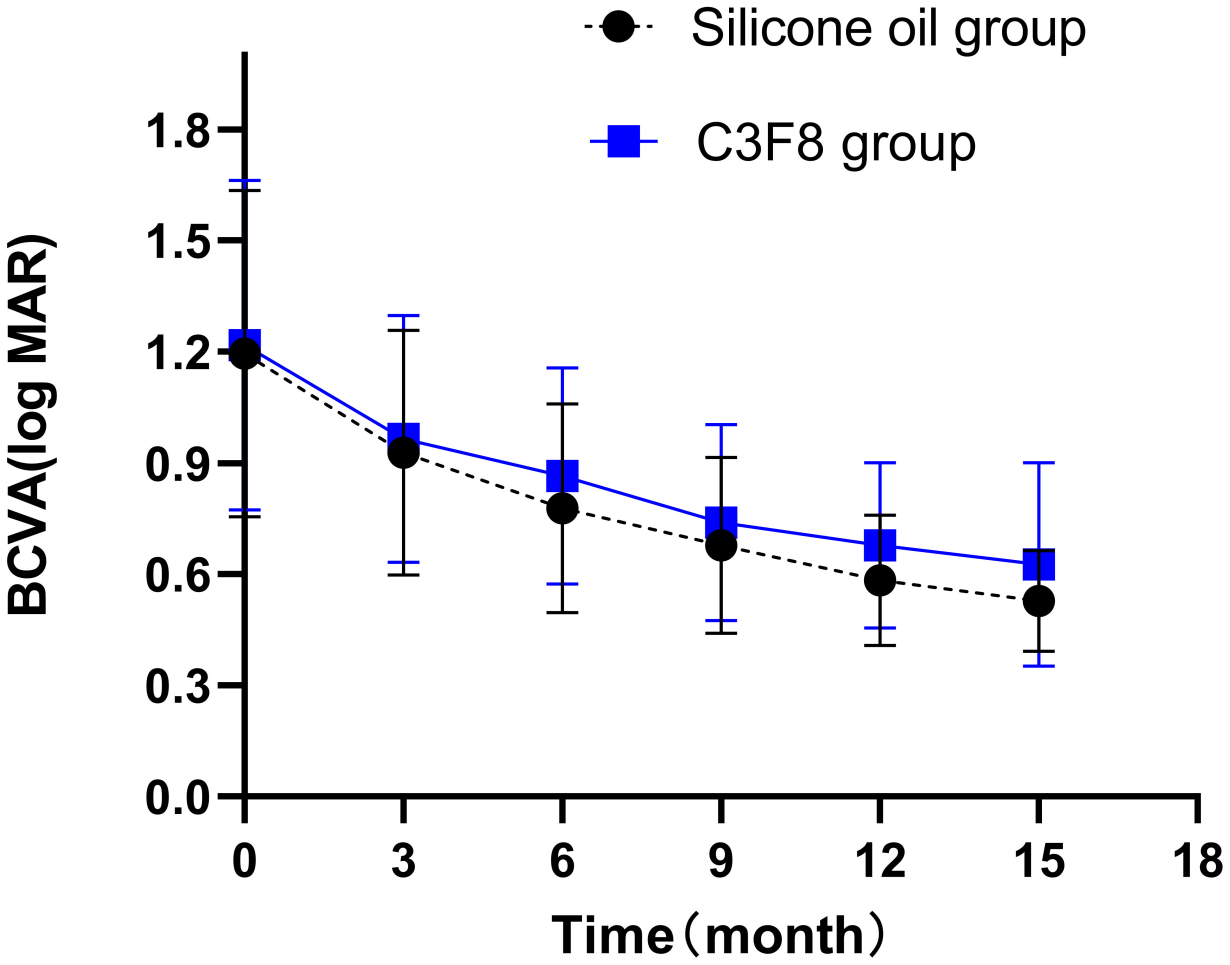
Postoperative best corrected visual acuity (BCVA) changes in the silicone oil and C3F8 groups. There was significant improvement in the BCVA of both groups. There was no statistically significant difference between both groups.

Both groups exhibited a statistically significant improvement in post-operative BCVA 15 months after the primary surgery (*P* < 0.05). There was no statistically significant difference in the BCVA between both groups (*P* > 0.05). The changes in pre-operative and post-operative BCVA are summarized in Supplementary Table 1 and displayed in Figure 3.

### 3.3 MaxFT change

**FIGURE 4:**
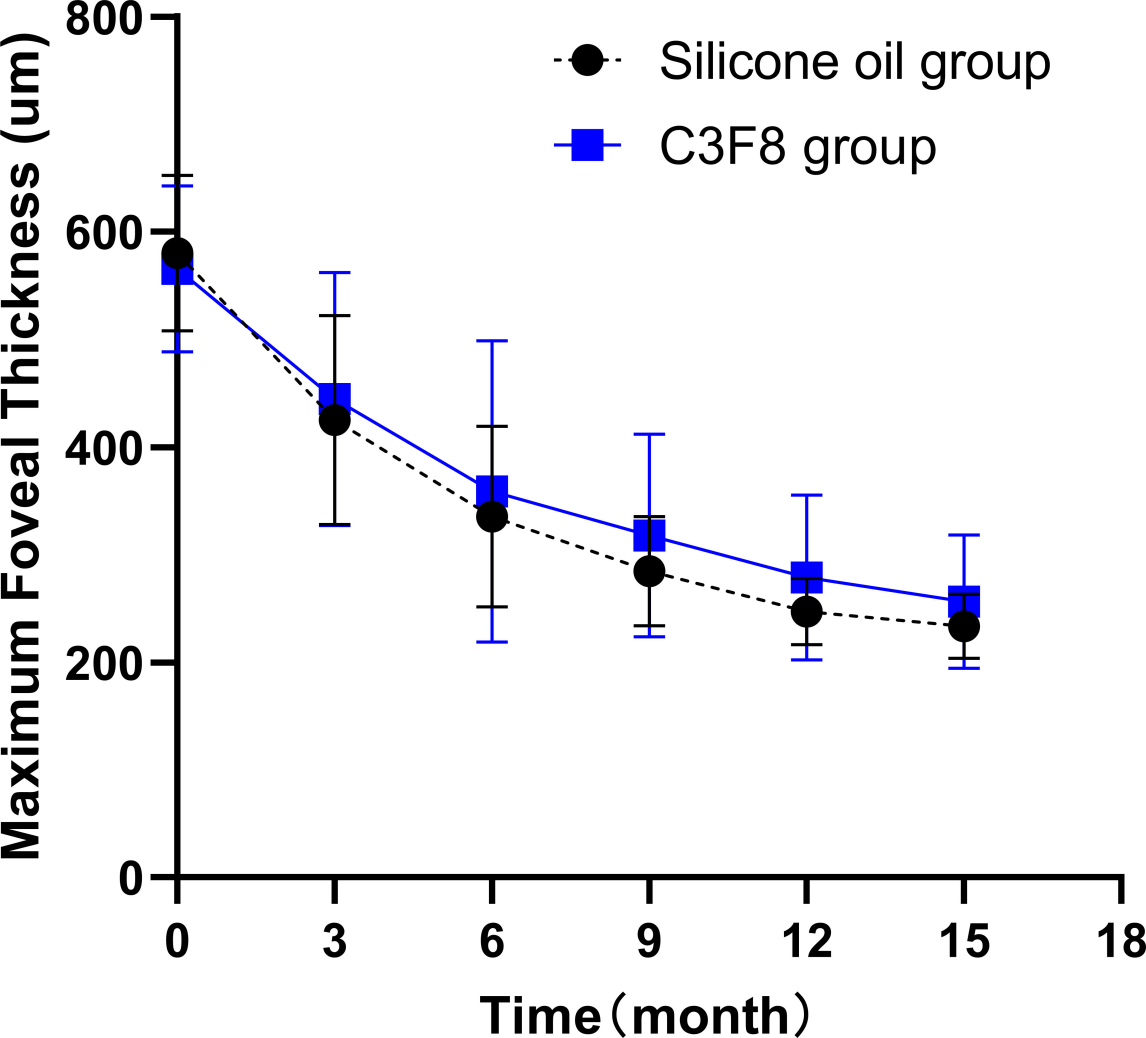
Postoperative maximum foveal thickness (MaxFT) changes in the silicone oil and C3F8 groups. There was significant reduction in the MaxFT of both groups. There was no statistically significant difference between both groups.

Both groups demonstrated a statistically significant reduction in post-operative MaxFT 15 months after the primary surgery (*P* < 0.05). There was no statistically significant difference in MaxFT between the two groups (*P* > 0.05). The changes in post-operative MaxFT are summarized in Supplementary Table 1 and displayed in Figure 4.

### 3.4 Recovery ratios

**FIGURE 5:**
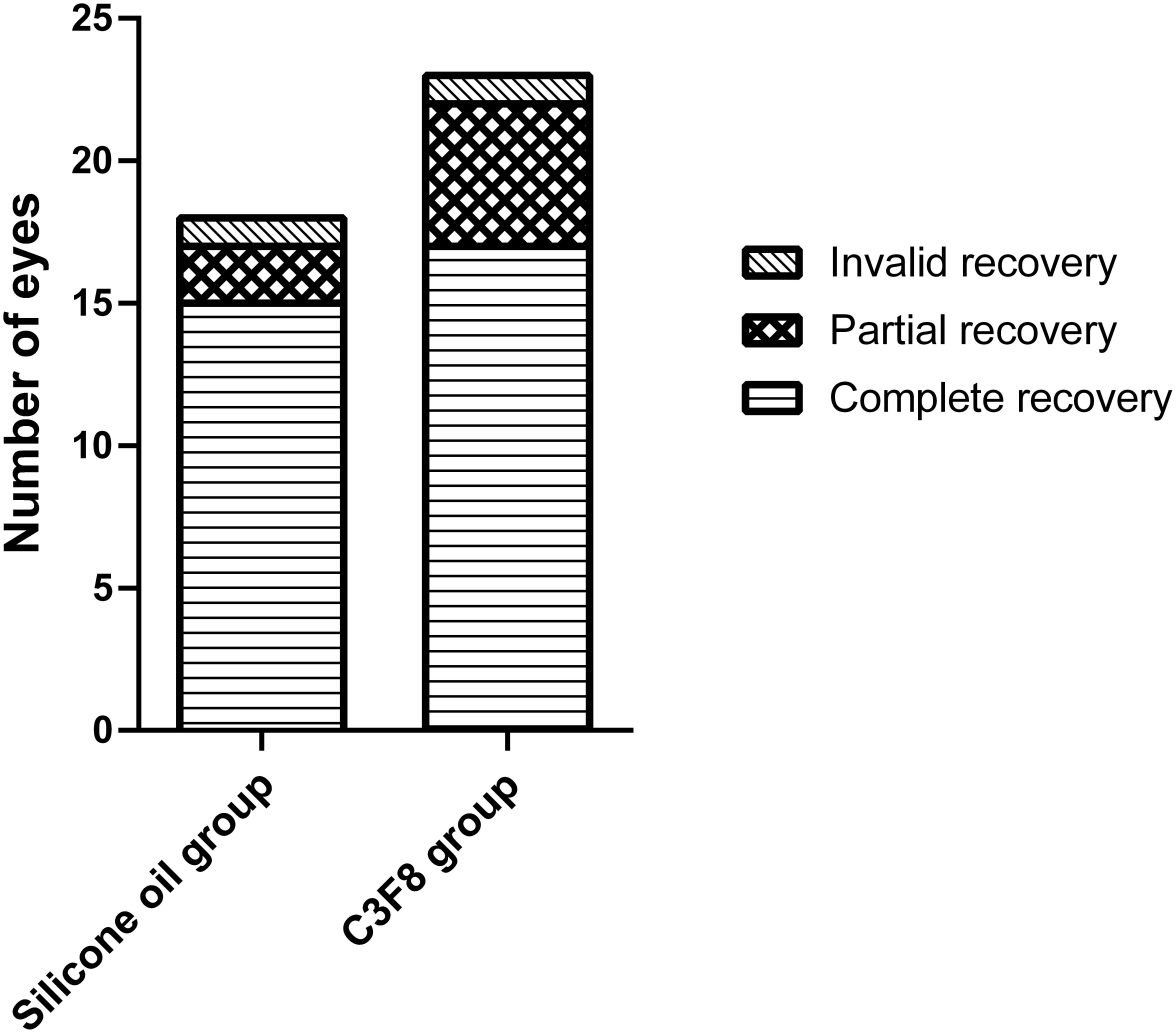
Recovery ratios of the silicone oil and C3F8 groups. There was no statistically significant difference in the recovery ratios between both groups.

At the final follow-up, the silicone oil group demonstrated complete, partial, and invalid recovery ratios of 83.33% (15/18 eyes), 11.11% (2/18 eyes), and 5.56% (1/18 eye), respectively. Comparatively, the C3F8 group demonstrated complete, partial, and invalid recovery ratios of 73.91% (17/23 eyes), 21.74% (5/23 eyes), and 4.35% (1/23 eye), respectively. There was no statistically significant difference in the recovery ratios between both groups (χ^2^ = 0.813, *P* = 0.666). The recovery ratios of the two groups are displayed in Figure 5.

### 3.5 Complication

Six eyes in the silicone oil group and three eyes in the C3F8 group developed transient ocular hypertension, which were responsive to topical intraocular pressure-lowering medications. One eye in the C3F8 group developed a MH. The right fundus and optical coherence tomography images of a patient with a postoperative MH are displayed in Supplementary Figure 1. No other complications, such as endophthalmitis, retinal detachment, fundus hemorrhage, or silicone oil emulsification, were noted. None of the patients in the silicone oil group developed myopic foveoschisis or foveal detachment recurrence after silicone oil removal.

## 4 Discussion

Our study showed that BCVA and MaxFT in both groups improved significantly after surgery. There was no significant difference in these values between both groups post-operatively. These results were similar to previous studies (17–20). While there was no statistically significant difference in the recovery ratios between both groups, the complete recovery ratio in the silicone oil group was higher than that in the C3F8 group during the final follow-up. This discrepancy may be due to our small sample size and short follow-up period. As such, future studies can improve on our data by including larger sample sizes and longer follow-up periods. Phacoemulsification with intraocular lens implantation was performed in all but two eyes. As such, there was a general improvement in BCVA. However, the improvement in BCVA may also be due to the resolution of MFFD. Lee et al. analyzed 32 patients with myopic foveoschisis who underwent vitrectomy and suggested that better preoperative BCVA and absence of foveal detachment were associated with better postoperative visual prognosis (21). Comparatively, Lehmann et al. proposed that preoperative BCVA was the only independent factor that influenced final BCVA, while the relationship between foveal morphology and vision was more complex (22). The study also showed that better preoperative BCVA was associated with better postoperative visual acuity. Age, preoperative MaxFT, and other factors showed no correlation (23). Several other prognostic factors have been identified in other studies. These include the location and extent of myopic foveoschisis and the presence of MH, ellipsoidal band rupture, or photoreceptor detachment in the fovea (24). One eye in the C3F8 group in this study developed an MH. Preoperatively, this eye was noted to have a severe posterior staphyloma. We theorized that the pathologically thinned retina in this eye contributed to the persistence of MFFD and the eventual formation of the MH.

The classic surgical treatment for MFFD include vitrectomy, posterior vitreous detachment, and removal of the posterior vitreous cortex of the macula (25). Additional steps, such as ILM peeling with or without foveal ILM sparing and tamponade with silicone oil or C3F8, increase the likelihood of visual recovery. However, the worst outcomes are associated with development of a full thickness MH or macular retinal detachment in the postoperative period.

The choice of intraocular tamponade material depends on the severity of MFFD. However, the need and type for intraocular tamponade in MFFD remains controversial (14). Many studies have shown that vitrectomy with long-acting gas tamponade, such as C3F8, can shorten retinal recovery time in patients with MFFD (26, 27). However, a different study demonstrated that the time needed to achieve anatomic macular reattachment exceeded the lifespan of the injected gas (28). As such, some patients may need longer durations of increased intraocular pressure to resolve MFFD. For this reason, silicone oil may be a better tamponade material overall. Long-term tamponade with silicone oil may slowly relieve the surface tension of a rigid ILM and reduce the possibility of future MH formation. Yao et al. proposed that vitrectomy with silicone oil tamponade without ILM peeling may be a good approach for MFFD, because it is simple and effective in preventing MH formation and macular hole retinal detachment. Silicone oil has less surface tension than gas. As such, it can provide a constant force against the surface of the retina (29). Retinal recovery is a slow process, and it is necessary to provide long-term tamponade, especially for severe cases of myopic foveoschisis. The long-term tamponade of silicone oil may be a desirable solution in patients with MFFD. Mancino et al. further demonstrated that silicone oil was a good option for patients with recurrent MFFD. All the patients in this study achieved complete anatomical recovery (30). Alkabes et al. demonstrated a long recovery period for MFFD and implicated that gas tamponade was inadequate in providing stable, long-term retinal pressure. Similar to Mancino, Alkabes suggested that a long-term tamponade material like silicone oil may be the better alternative (31). Silicone oil tamponade is associated with some complications, such as secondary glaucoma, progressive cataract, silicone oil emulsification, and unexplained visual loss. However, most complications can be treated with medication or surgery. The incidence of unexplained vision loss during silicone oil tamponade or after silicone oil removal is 1%–30% (32). The etiology of unexplained vision loss is still unclear. Possible pathophysiology may be silicone oil-related macular structural change, emulsification, phototoxicity and dissolution of fat-soluble lutein and zeaxanthin (33). These complications except silicone oil emulsification and unexplained visual loss may also occur with long-acting gas, such as C3F8, tamponade.

Internal limiting membrane peeling as a treatment for MFFD is also controversial. The ILM supplies the main traction forces on the posterior pole of the retina. ILM peeling removes these traction forces, which allows the retina to better fit the posterior pole. This effect is even more pronounced in patients with posterior scleral staphylomas (34). Many studies have shown that ILM peeling is effective (35, 36). Mao et al. demonstrated that vitrectomy with ILM peeling resulted in better postoperative anatomy and vision compared to vitrectomy alone (37). However, the retina of patients with MFFD is very thin. ILM peeling may induce MH formation, which is a significant complication (38, 39). Other studies proposed that choroid thickness may be a bigger determinant of MH formation than ILM manipulation (40). In the study by Peng et al., 32 eyes with myopic foveoschisis underwent vitrectomy with standard ILM peeling. No MH was noted over a 3-years follow-up period (41). In the study by Qi et al., patients underwent vitrectomy without ILM peeling. Six cases (5%) of MH were noted during the follow-up period (42).

Macular hole formation is a significant complication. As such, some scholars have proposed to preserve the foveal ILM during ILM peeling. This facilitates the structural and functional recovery of the fovea, as well as reduces the risk for MH formation (43, 44). This study also suggested that postoperative anatomic and visual outcomes following foveal-sparing ILM peeling may be comparable to those after standard ILM peeling. However, fovea-sparing ILM peeling may be superior by reducing the risk for MH formation, which ultimately improves post-operative BCVA (45). Shiraki et al. (40) examined 26 and 76 eyes that underwent fovea-sparing ILM peeling and standard ILM peeling, respectively. The study found no statistically significant difference in postoperative visual acuity and recovery times between both groups (40). Other studies have detected a slightly higher risk for anterior retinal membrane recurrence in eyes that underwent fovea-sparing ILM peeling, which may limit the effectiveness of fovea-sparing ILM peeling (46, 47).

Our study has some limitations. First, the study was a retrospective, single-center study. Second, the study involved a small group of patients. Third, our study covered a relatively short follow-up period of 12 months. Future studies may benefit from following a randomized controlled study design that covers a larger sample size and longer follow-up period over multiple centers.

In conclusion, pars plana vitrectomy and fovea-sparing ILM peeling with silicone oil or C3F8 tamponade have similar visual and structural benefits. Both options provide similar structural and functional outcomes without increasing the risk for postoperative complications. As such, both may be considered as safe and effective treatments for MFFD.

## Data Availability

The raw data supporting the conclusions of this article will be made available by the authors, without undue reservation.

## Supplementary material

Supplementary Figure 1 Right fundus and optical coherence tomography images of a representative patient with a postoperative macular hole from the C3F8 group. **(a)** Preoperative fundus images showing retinal and choroidal atrophy in a highly myopic patient; **(b)** preoperative optical coherence tomography images showing outer and inner myopic foveoschisis, foveal detachment, and an outer macular lamellar hole; **(c)** postoperative fundus images 3 months after vitrectomy with C3F8 tamponade and phacoemulsification with intraocular lens implantation showing retinal and choroidal atrophy; **(d)** postoperative optical coherence tomography images showing complete resolution of retinal foveoschisis and foveal detachment but with a large full-layer macular hole.
