# Supplementary figures and images for: Comparison of the effectiveness of vitrectomy with silicone oil or perfluoropropane tamponade for myopic foveoschisis with foveal detachment
Source: Front Med (Lausanne). 2025 Sep 23;12:1602386. doi: 10.3389/fmed.2025.1602386 (PMC12500636; doi:10.3389/fmed.2025.1602386)

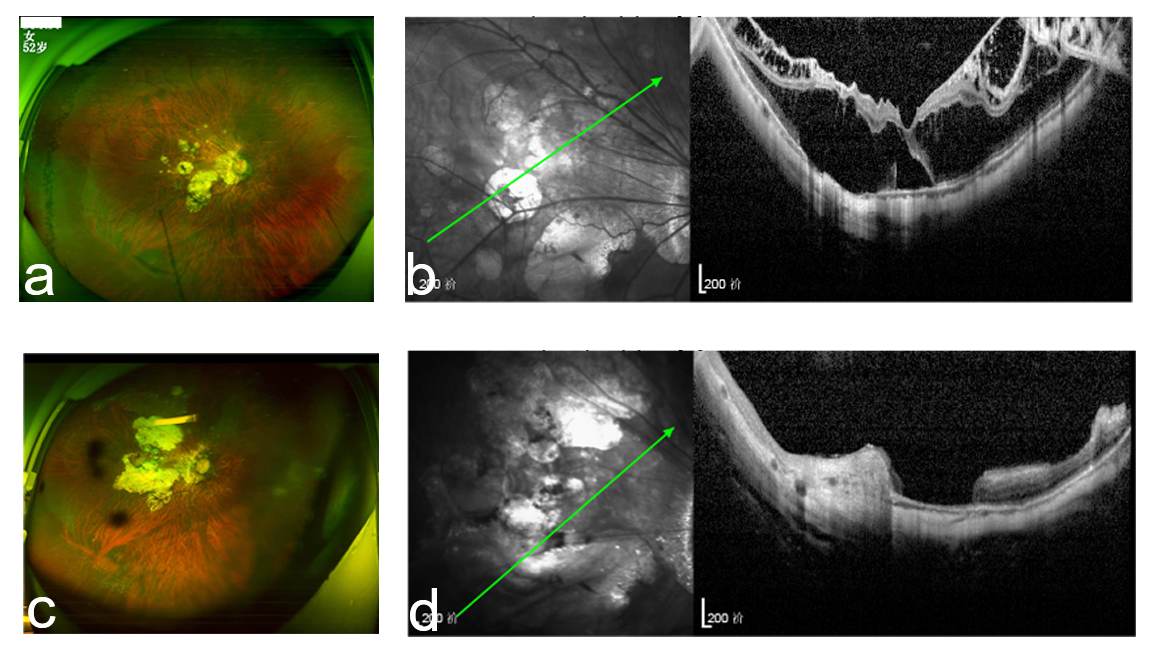

Supplement: Supplementary Figure 1 — Right fundus and optical coherence tomography images of a representative patient with a postoperative macular hole from the C3F8 group. (a) Preoperative fundus images showing retinal and choroidal atrophy in a highly myopic patient; (b) preoperative optical coherence tomography images showing outer and inner myopic foveoschisis, foveal detachment, and an outer macular lamellar hole; (c) postoperative fundus images 3 months after vitrectomy with C3F8 tamponade and phacoemulsification with intraocular lens implantation showing retinal and choroidal atrophy; (d) postoperative optical coherence tomography images showing complete resolution of retinal foveoschisis and foveal detachment but with a large full-layer macular hole. [file Image_1.tif]
