# Supplementary material for: Comparison of the effectiveness of vitrectomy with silicone oil or perfluoropropane tamponade for myopic foveoschisis with foveal detachment
Source: Front Med (Lausanne). 2025 Sep 23;12:1602386. doi: 10.3389/fmed.2025.1602386 (PMC12500636; doi:10.3389/fmed.2025.1602386)
Supplement: Supplementary file 2 [file Table_1.docx]

Supplementary Table 1. Change in preoperative and postoperative best corrected visual acuity and maximum foveal thickness in the two groups

|  | | Silicone oil group (18 eyes) | C3F8 group  (23 eyes) | Test value | P value |
| --- | --- | --- | --- | --- | --- |
| BCVA (LogMAR) | V0 | 1.19±0.441 | 1.22±0.445 | T=0.165 | 0.870 |
|  | V1 | 0.928±0.330 | 0.965±0.332 | T=0.359 | 0.722 |
|  | V2 | 0.778±0.282 | 0.865±0.292 | T=0.966 | 0.340 |
|  | V3 | 0.678±0.237 | 0.739±0.264 | T=0.772 | 0.445 |
|  | V4 | 0.583±0.176 | 0.678±0.224 | T=1.48 | 0.147 |
|  | V5 | 0.528±0.136 | 0.626±0.275 | T=1.39 | 0.174 |
|  | Test value | F=13.54 | F=11.3 | NA | NA |
|  | P value | <0.0001 | <0.0001 | NA | NA |
| MaxFT  (um) | V0 | 580±72.1 | 565±77.2 | U=181.5 | 0.5112 |
|  | V1 | 425±97.3 | 445±117 | U=188 | 0.6167 |
|  | V2 | 335±83.8 | 359±140 | U=162 | 0.2425 |
|  | V3 | 285±50.8 | 318±94.2 | U=148 | 0.1375 |
|  | V4 | 247±31.1 | 279±76.4 | U=126 | 0.0851 |
|  | V5 | 233±30.1 | 256±61.9 | U=109 | 0.0624 |
|  | Test value | F=79.71 | F=32.3 | NA | NA |
|  | P value | <0.0001 | <0.0001 | NA | NA |

Abbreviations: BCVA, best corrected visual acuity; MaxFT, maximum foveal thickness; NA, not available. V0, pre-operation (< 2 weeks) of Silicone oil or C3F8 tamponade; V1, 3 month (± 2 weeks) of post-operation of Silicone oil or C3F8 tamponade; V2, 6 month (± 2 weeks) of post-operation of Silicone oil or C3F8 tamponade; V3, 9 month (± 2 weeks) of post-operation of Silicone oil or C3F8 tamponade; V4, 12 month (± 2 weeks) of post-operation of Silicone oil or C3F8 tamponade; V5, 3 month (± 2 weeks) of post-operation of Silicone oil removal or 15 month (± 2 weeks) of post-operation of C3F8 tamponade.
